# Barriers and facilitators of adherence to clinical practice guidelines in Germany—A systematic review

**DOI:** 10.1111/jep.14173

**Published:** 2024-10-16

**Authors:** Eni Shehu, Charlotte M. Kugler, Niklas Schäfer, Diane Rosen, Corinna Schaefer, Thomas Kötter, Markus Follmann, Dawid Pieper

**Affiliations:** ^1^ Brandenburg Medical School (Theodor Fontane), Institute for Health Services and Health Systems Research Faculty of Health Sciences Brandenburg Rüdersdorf Germany; ^2^ Brandenburg Medical School (Theodor Fontane) Center for Health Services Research Rüdersdorf Germany; ^3^ Brandenburg Medical School Theodor Fontane JBI Affiliated Group EBB Brandenburg an der Havel Germany; ^4^ Brandenburg Medical School (Theodor Fontane) Neuruppin Germany; ^5^ Clinic for Otorhinolaryngology Hennigsdorf Germany; ^6^ Department of Health Alice Salomon University of Applied Sciences Berlin Germany; ^7^ German Agency for Quality in Medicine Berlin Germany; ^8^ Institute of Family Medicine University Medical Centre Schleswig‐Holstein Lübeck Germany; ^9^ German Guideline Program in Oncology, German Cancer Society Berlin Germany

**Keywords:** clinical practice guideline, enabling factors, evidence‐based medicine, guideline adherence, health services research, systematic review

## Abstract

**Rationale:**

Clinical Practice Guidelines (CPGs) represent evidence‐based tools designed to assist healthcare practitioners and patients in decisions in clinical practice. Evidence supports the clinical benefits of adhering to CPGs. However, their successful implementation and adherence in clinical settings often encounter challenges.

**Aims and Objectives:**

This systematic review aimed to explore barriers and facilitators influencing adherence to CPGs in Germany.

**Method:**

The protocol of this study was registered in the Open Science Framework (OSF) registry (DOI: 10.17605/OSF. IO/GMFUB). In November 2022 we searched on PubMed and Embase for primary studies employing qualitative, quantitative and mixed‐methods approaches that focus on barriers or facilitators to CPGs adherence in the Germany. Two reviewers independently screened articles, extracted data, and evaluated the quality of the studies. The collected data on barriers and facilitators of CPG adherence were systematically categorized and analyzed using the Theoretical Domains Framework (TDF).

**Results:**

A total of 24 studies were included, mainly focusing on adherence to national CPGs. This review introduces a new domain, *guideline characteristics*, reflecting the need to address barriers and facilitators to CPG development, implementation, dissemination and format, which couldn't be encompassed within the existing 14 domains of TDF framework. Among healthcare professionals, the most frequently reported influencing factors were related to *the environmental context and resources* (encompassing aspects such as employer support for CPG utilization), *the CPG development and dissemination process* (including layout, wording, and interactive tools) and *beliefs about consequences* (such as contradictions with practical experience). *Knowledge* (knowledge about the content of CPGs, awareness about published CPGs), primarily as a barrier, and *reinforcement* facilitators (notably financial support), were also frequently reported.

**Conclusion:**

The findings revealed multilevel factors contributing to CPG adherence, with environmental context and resources emerging as the most frequently reported considerations. This systematic review offer holistic insights into the barriers and facilitators of CPG adherence in Germany. The results contribute to a better understanding of the topic and serve as a resource for developing targeted strategies to enhance CPG adherence and implementation within the German healthcare system.

**Abbreviations**
AWMF: The German Association of the Scientific Medical Societies
ÄZQ: German Agency for Quality in Medicine
CPGs: Clinical practice guidelines
DEGAM: German Society of General Practice/Family Medicine
DKG: German Hospital Federation
EbM: Evidence based Medicine
GPs: General practitioners
MMAT: Mixed Methods Appraisal Tool
PTs: Physical therapists
TDF: The Theoretical Domains Framework

## 1 INTRODUCTION

Clinical practice guidelines (CPGs), as defined by Institute of Medicine (2011), are “*statements that include recommendations intended to optimize patient care that are informed by a systematic review of evidence and an assessment of the benefits and harms of alternative care options*”.^1^ They tackle clinical issues, providing guidance on managing clinical conditions or symptoms, and are commonly aimed for use by health care providers and clinic managers. The scope of CPGs encompasses all healthcare practice areas involved in monitoring, screening, diagnosing, management of clinical conditions. The use of CPGs has shown various benefits, such as improved clinical and survival outcomes, reduced length of hospitalisation, and reduced health‐care costs.^2^, ^3^, ^4^, ^5^ However, evidence suggests that the CPGs adherence in clinical practice may be poor.^6^, ^7^, ^8^, ^9^, ^10^ In the German context, one study found that 38.2% of cases of unstable angina were managed in accordance with CPGs.^11^ Furthermore, another study revealed full adherence to CPGs among physiotherapists in 17.2% of hip osteoarthritis management cases, and in 8.6% for knee osteoarthritis management.^12^ To best of our knowledge, there hasn't been a comprehensive examination of the rate of CPGs adherence across Germany.

An adequate analysis of the barriers that prevent healthcare providers from using CPGs in practice has demonstrated to be an important initial step in improving CPGs adherence and, subsequently, quality of care.^13^, ^14^ For instance, through identifying and analyzing barriers and facilitators, stakeholders involved in the development and implementation of CPGs gain valuable insights, pinpointing areas requiring improvement or attention. This information can then contribute to the conceptualisation of more successful and expedited strategies for CPG uptake. Barriers and facilitators can differ depending on the local or regional context. An exploration of the barriers and facilitators to a regional context is important, since factors related to cultural features, the work climate and health system organisation might impact the adherence of CPGs in clinical practice.^15^ For example, the results of a systematic review that explored the adherence to national antibiotic guidelines for community‐acquired pneumonia in Australia, showed that the influence of senior doctors on junior doctors impacted CPG adherence, since prescribing was largely under the direction of senior doctors.^16^ To our knowledge, no systematic review explored the barriers and facilitators of adherence to CPGs in the German context, yet.

Exploration of barriers and facilitators for the CPG adherence is the first step into improving the usage of CPG in clinical practice and increasing uptake.^17^ Different implementation theoretical models and frameworks are available for offering guidance in effective implementation and sustainability of the research results.^18^, ^19^ The theoretical domain framework (TDF) is an implementation framework, originating from 33 behaviour change theories and 128 theoretical constructs, which are synthetized into 14 validated domains. This framework aims to provide a comprehensive theoretical assessment of implementation problems.^20^ Many authors have used TDF in their implementation projects, and several of them focused on topics regarding CPGs.

The aim of this systematic review was to investigate the barriers and facilitators regarding the adherence to CPGs in Germany, with the help of TDF. The CPGs included and analyzed in this review encompass various clinical practice areas from different medical specialities, healthcare disciplines and clinical settings.^21^ The results of this systematic review give an overview regarding the facilitators and barriers, which could serve as a basis for future improvement strategies for CPGs adherence.

## 2 METHODS

We carried out a systematic literature research to explore the barriers and facilitators to CPGs in Germany. The protocol of this study was registered in the Open Science Framework (OSF) registry.^22^ We adhered to the Preferred Reporting Items for Systematic Reviews and Meta‐Analyses (PRISMA) in the preparation of this manuscript, where appropriate.^23^ The text for the method section was recycled from the protocol published in the OSF in accordance with the guidance provided by the Text Recycling Research Project.^24^

### 2.1 Eligibility criteria

We included articles that: (1) were primary studies; (2) participants were health professionals (e. g. doctors, nurses, therapists) or decision‐makers (e.g. hospital managers) in Germany; (3) reported on barriers or facilitators of CPGs adherence of all types of German or international CPGs from all specialty areas and context; (4) included data from Germany (national or multinational studies, if data for Germany was reported separately); (5) were published in German or English language; and (6) were published after 01 January 2011. We limited the search to studies published after 2011 because the influencing factors might have changed over time. We considered studies that examine different CPGs, studies that examine only one CPG, or those that examine only one component or individual recommendations of a CPG.

It should be noted that adherence to CPGs can also mean deviating from a recommendation for good reasons (such as age, comorbidities, non‐correspondence of the recommendations with the patients' wishes etc.).^25^, ^26^ Therefore, the term “guideline adherence” in this review refers to the consistency between clinicians' actions and recommendation of the CPGs for cases where deviations from CPG recommendations are not justified.

### 2.2 Information sources and search strategy

We searched in PubMed and EMBASE databases to identify relevant studies. Additional searches were conducted in Google Scholar and manually by cross‐checking the reference lists of all included primary studies and by hand searching available abstracts from reports of conferences from the German Network for Health Services Research and the German Network for Evidence‐based Medicine e.V. Furthermore, we contacted stakeholders for additional studies. The comprehensive search strategy is provided in Supporting Information S1: Material S1. The search took place in November 2022.

### 2.3 Selection process

Three co‐authors of this study performed the screening and data extraction process in pairs of two (Eni Shehu, Charlotte M. Kugler, Niklas Schäfer). First, titles and abstracts were screened by two authors independently in Rayyan.^27^ The full texts of the included abstracts were then reviewed by two independent authors against the inclusion criteria. Papers were included only when both reviewers considered the article as eligible. Discrepancies were resolved by discussion. If necessary, a senior researcher (Dawid Pieper) was involved.

### 2.4 Data extraction process

Descriptive data (first author, publication year, study aim, study type, place, funding, CPG topics, CPG type, year of data collection, number of participants, inclusion criteria, participants' characteristics, speciality, clinical setting, methods of data collection, data analysis, framework) were extracted by one reviewer (Eni Shehu) via Microsoft Excel (Microsoft Corporation) and checked by a second reviewer (Diane Rosen).

Two independent reviewers extracted the reported barriers and facilitators from each study via a standardised data abstraction form in Excel (Eni Shehu, Charlotte M. Kugler, Niklas Schäfer). Then, one reviewer compared both versions for completeness and accuracy. Any discrepancies were resolved by discussion, or if necessary, a third reviewer was involved. In case of any uncertainty, authors of the primary studies were contacted to request missing or additional data for extracted data items.

### 2.5 Methodological quality in individual studies

To assess the methodological quality of included studies, we used the Mixed Methods Appraisal Tool (MMAT).^28^ Two reviewers (Eni Shehu and Charlotte M. Kugler) independently assessed the included studies. In compliance with the recommendations of MMAT, questions were answered for each section with ‘yes,’ ‘no’ or ‘cannot tell’ but no total score was generated. In the case of discrepancies, consensus was sought through discussion.

### 2.6 Data synthesis and analysis

Data collected on barriers and facilitators that impact the adherence to CPGs in Germany were mapped and analyzed based on the domains of the TDF.^20^, ^29^ We followed the guidelines of Akties et al. when using the TDF framework.^30^ An additional domain named *guideline characteristics* was added in this review, due to the high number of the barriers and facilitators reported that were related to the CPGs themselves (developments process, implementation and dissemination process, format etc.) and could not be categorized in any of TDF domains. Two independent reviewers mapped the barriers and facilitators to the 14 domains (Eni Shehu and Charlotte M. Kugler or Eni Shehu and Niklas Schäfer). In case of discrepancies, discussions took place and consensus was sought.

The different influencing factors in each TDF domain were then subcategorized into theoretical constructs of TDF by one reviewer (Eni Shehu), and controlled by another reviewer (Charlotte M. Kugler). In case of discrepancies, discussions took place and consensus was sought.

### 2.7 Stakeholder involvement

Stakeholder involvement was particularly important to our systematic review, enabling us to consider the valuable perspectives of professionals engaged in the development and implementation of CPGs in Germany.^31^ Experts from German Agency for Quality in Medicine (ÄZQ), German Guideline Program in Oncology, and German Society of General Practice/Family Medicine (DEGAM) were involved in this systematic review. Specifically, they were involved in the development of the protocol, in study selection, discussion of the results and in critically reviewing the final manuscript.

## 3 RESULTS

**Figure 1:**
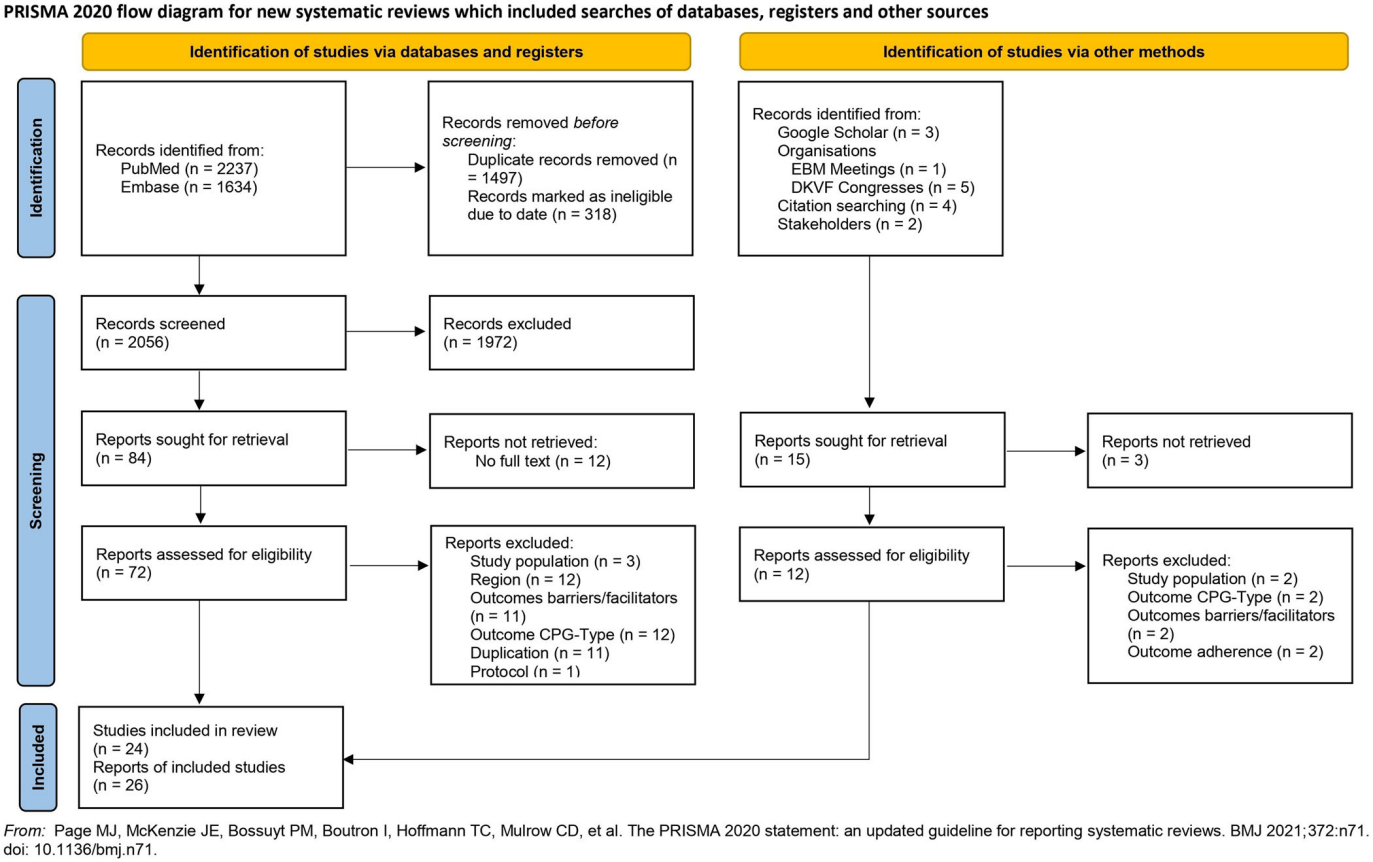
PRISMA flow diagram of studies' screening and selection. PRISMA, Preferred Reporting Items for Systematic Reviews and Meta‐Analyses.

There were 24 studies (26 reports) included in this systematic review.^32^, ^33^, ^34^, ^35^, ^36^, ^37^, ^38^, ^39^, ^40^, ^41^, ^42^, ^43^, ^44^, ^45^, ^46^, ^47^, ^48^, ^49^, ^50^, ^51^, ^52^, ^53^, ^54^, ^55^, ^56^, ^57^ The flow chart is provided in Figure 1.

### 3.1 Study characteristics

Table 1 provides an overview of the characteristics of the 24 studies included in this review. Five studies were qualitative (conducted as focus groups and/or individual‐ interviews),^36^, ^48^, ^50^, ^54^, ^55^ eighteen studies were quantitative (surveys)^33^, ^34^, ^35^, ^37^, ^38^, ^39^, ^40^, ^41^, ^42^, ^43^, ^44^, ^45^, ^46^, ^47^, ^49^, ^51^, ^52^, ^53^, ^56^, ^57^ and one study used a mixed‐methods design.^32^ The sample sizes ranged from nine to 1,361 individual participants. Notably, two studies reported solely the number of participating clinics or departments.^45^, ^57^

**Table 1:** Description table of included studies.

Nr	First author, year	Aim	Study type (methods of data collection)	Place	CPG topic	Participants included in analysis N (mode)	Clinical setting	Data analysis
1	Schwarz et al.^32^	To explore the awareness and applicability of clinical practice guidelines (CPGs) and identify potential for improvement.	Mixed‐methods (survey and interviews).	Germany, nationwide.	All German CPGs developed from “The Program for National Health Care Guidelines (NVL).”	558 (surveys) and 46 (interviews).	Not specified.	Descriptive and qualitative content analysis.
2	Bahns et al.^33^	To evaluate the current physiotherapy management of patients with low back pain, and to explore CPG adherence.	Quantitative (survey).	Germany, nationwide.	Low back pain.	1,361	Outpatient, inpatient, rehabilitation clinic.	Exploratory univariate logistic regression.
3	Bannow et al.^34^	To investigate the acceptance and practical implication of a CPG one year after their publication.	Quantitative (survey).	Schleswig‐Holstein, Germany.	Management of the arterial hypertonia.	259	Outpatient.	Descriptive statistics.
4	Brenner et al.^35^	To capture the physician perception of CPG adherence and leverage points for increasing adherence.	Quantitative (survey).	Germany, nationwide.	Type 2 diabetes mellitus.	46	Outpatient and inpatient.	Descriptive statistics.
5	Freier et al.^36^	explore the general practitioners' perspective on prescribing, medical non‐adherence as well as their attempts to improve adherence.	Qualitative (interviews).	Berlin and Brandenburg, Germany.	Myocardial infarction, long‐ term care.	16	Outpatient.	Thematic framework.
6	Gaigl et al.^37^	To examine the implementation status of the current and international representative German evidence‐ and consensus‐based CPGs.	Quantitative (survey).	Germany, nationwide.	Schizophrenia and psychosocial therapies.	657	Inpatient, outpatient, research, setting across subgroups, etc.	Descriptive statistics, chi test, t‐tests, and ANOVA.
7	Hoffmann^38^, ^39^	To evaluate the use and implementation of the CPGs in Neonatal intensive care units and hospital pharmacies.	Quantitative (survey).	Germany, nationwide.	Nutrition.	186	Inpatient.	Descriptive statistics.
8	Kalies et al.^40^	Explore the professionals' willingness to adopt already existing recommendations on palliative care.	Quantitative (survey).	Germany, nationwide.	Palliative care.	1031	Outpatient, hospice, inpatient, research.	Logistic regressions.
9	Karbach et al.^41^	To investigate the link between primary care physicians' knowledge of and compliance with CPGs in the primary care.	Quantitative (survey).	Saxony and North Rhine, Germany.	Arterial hypertension, heart failure, and chronic coronary heart disease.	1121	Primary care.	Descriptive statistics, chi square.
10	Kranz et al.^42^, ^43^	To evaluate the potential reasons for CPG violence.	Quantitative (survey).	Germany and Austria.	Noncomplicated urinary tract infections.	563 from Germany.	Inpatient and outpatient.	Descriptive.
11	Lapillonne et al.^44^	To evaluate compliance and determine factors that influence compliance to CPGs.	Quantitative (survey).	Germany, United Kingdom, France and Italy.	Parenteral nutrition in preterm infants.	54	Inpatient.	Analysis of null hypothesis ‘No pairwise differences in proportions across subgroups’‐χ² tests.
12	Laux et al.^45^	How often drug monitoring is used.	Quantitative (survey).	Germany, nationwide.	Therapeutic drug monitoring in psychiatry.	31 clinics.	Inpatient.	Descriptive analysis.
13	Lech et al.^46^	To examine the role of a specific CPG in primary care.	Quantitative (survey).	Berlin and Brandenburg, Germany.	Unipolar depression.	85	Outpatient.	Multiple logistic regression.
14	Lohmann et al.^47^	In which area physicians followed or did not implement the CPGs.	Quantitative (survey).	Germany, nationwide.	Dementia.	329	Outpatient.	Descriptive analysis and Bonferonni.
15	Muehlhaeuser et al.^48^	To determine the acceptance and practicability of a CPG.	Qualitative (focus groups or interviews).	Lübeck, Germany.	Multi‐morbidity.	9	Outpatient.	Content analysis.
16	Ostermann et al.^49^	To verify the implementation of a specific recommendation of CPG.	Quantitative (survey).	Germany, nationwide.	Non‐small cell lung cancer.	94	Inpatient and outpatient.	Not described.
17	Peters‐Klimm et al.^50^	To conduct a barrier and needs analysis.	Qualitative (focus group).	Heidelberg, Germany.	Chronic heart failure.	13	Outpatient.	Content analysis.
18	Scheffler et al.^51^	To describe self‐reported adherence to a CPG and explore the barriers against facilitators of guideline use.	Quantitative (survey).	Germany, nationwide.	Rehabilitation of mobility after stroke.	97	Mainly rehabilitation center and outpatient.	Descriptive statistics.
19	Schielein et al.^52^	To identify possible concerns and barriers of treatment according to CPGs.	Quantitative (survey).	Bavaria, Germany.	psoriasis and chronic spontaneous urticarial.	137	Outpatient.	The barrier subdivision.
20	Schmieder et al.^53^	To gain insight into the situation, including the attitude of physicians towards CPG.	Quantitative (survey).	12 countries.	Cardiovascular risk prevention and management.	66 in Germany.	Not specified.	Descriptive statistics.
21	Stephan et al.^54^	To understand which challenges and barriers PCPs see when diagnosing and which facilitators and barriers may arise with respect to CPG introduction.	Qualitative (interviews).	Bavaria, Germany.	Vertigo management.	12	Outpatient.	Inductive and deductive (via “Capability Opportunity Motivation‐Behavior model).
22	Tiedje et al.^55^	To develop a computerized decision aid and to clarify whether it reduces patients' decision conflict.	Qualitative (focus groups).	Muenster, Germany.	Prostate screening in urological practices.	22	Outpatient.	Qualitative content analysis.
23	Traidl et al.^56^	To explore and describe different approaches to identify current challenges when managing patients.	Quantitative (survey).	German speaking countries.	Atopic dermatitis.	358 in Germany.	Inpatient and outpatient.	Descriptive statistics.
24	Westhoff et al.^57^	To overview the use of techniques with focus on the content of the CPGs.	Quantitative (survey).	Within 50 km from Hemer, Germany.	Noninvasive respiration in acute respiratory insufficiency.	63 departments.	Inpatients.	Descriptive analysis.

Abbreviation: CPG, clinical practice guidelines.

Fifteen studies used closed questions to explore the barriers and/or facilitators, and eight studies used open‐ended questions. In two studies participants were not directly asked about barriers and/or facilitators but the available data was used to investigate compliance to CPGs with the influence of knowledge,^41^ and the number of beds effectively managed by the specialist disciplines.^57^ Among the 24 included studies, nine focussed solely on exploring barriers to CPG adherence,^32^, ^33^, ^34^, ^40^, ^44^, ^45^, ^46^, ^52^, ^53^ one exclusively delivered facilitators,^47^ and the remaining studies investigated both facilitators and barriers.

Overall, four studies were conducted internationally^42^, ^43^, ^44^, ^53^, ^56^ others were exclusively conducted in Germany. Within the Germany‐conducted studies, ten specifically targeted particular regions^34^, ^36^, ^41^, ^46^, ^52^, ^54^, ^57^ or cities.^48^, ^50^, ^55^ The majority of the studies explored adherence to CPG for cardiovascular diseases,^34^, ^36^, ^41^, ^50^, ^51^, ^53^ followed by psychiatry and psychosomatics,^37^, ^45^, ^46^, ^47^ neonatology,^38^, ^39^, ^44^ urology,^42^, ^43^, ^55^ pulmonology^49^, ^57^ and dermatology.^52^, ^56^ Other studies assessed adherence to CPGs for back pain,^33^ type 2 diabetes,^35^ palliative care,^40^ multimorbidity,^48^ and vertigo.^54^

Fifteen studies evaluated adherence to German CPGs, four examined adherence to an international CPG,^34^, ^38^, ^39^, ^44^, ^53^ while five focused on more than one CPG.^36^, ^37^, ^40^, ^41^, ^54^

### 3.2 Quality of the included studies

The common limitations within the qualitative analysis were the insufficient substantiation of the results by data, inadequate derivation of the results from the data, and the coherence between data sources, collection, analysis, and interpretation.

All quantitative analysis showed to have a high risk of nonresponse bias. Other limitations included nonrepresentative sample of the target population and nonappropriate measurements of barriers and facilitators. One study could not be fully evaluated due to missing information about the target group.^57^ A complete report of the assessed quality of the included studies can be found in supplementary material 2.

### 3.3 Barriers and facilitators of CPG adherence, categorized in TDF domains

In total, 138 barriers and 91 facilitators were identified. The breakdown of various barriers and facilitators for each TDF domain is presented in Table 2, while Table 3 provides a summary of those identified factors.

**Table 2:** The number of barriers and facilitators for each TDF domain.

Domain	Number of barriers	Number of facilitators	Number of studies
Knowledge	10	9	14
Skills	12	4	8
Social/professional role and identity	6	5	10
Beliefs about capabilities	2	1	3
Optimism	0	0	0
Beliefs about consequences	23	3	16
Reinforcement	6	5	7
Intentions	3	0	3
Goals	2	1	3
Memory, attention and decision process	2	5	4
Environmental context and resources	39	25	17
Social influences	3	3	5
Emotions	2	0	2
Behavior regulations	3	3	4
Guideline characteristics^a^	24	26	15

Abbreviation: TDF, Theoretical Domains Framework.

^a^ This domain was added additionally to the 14 domains of TDF.

**Table 3:** Summary of identified barriers and faciliators for each TDF domain.

Barriers	Facilitators
Knowledge	
*'Knowledge of task environment' construct* ‐ I do not know where I can find CPGs.^33^ ‐ No availability of the CPGs.^50^ ‐ Lack of knowledge from colleagues/other specialties.^42^, ^43^	*'Knowledge of task environment' construct* ‐ The journal where CPG is published.^42^, ^43^ ‐ Increased knowledge of the doctors.^54^
*'Procedural Knowledge' construct* ‐ (Doctors) required knowledge of specific recommendations.^51^ ‐ It is hard to keep track of the recommendations.^36^ ‐ (Physical therapists) were unaware of the link between the CPG and the phases of neurorehabilitation in Germany.^51^	*'Procedural Knowledge' construct* ‐ Creation of an increased awareness about the advantages of CPGs (e.g. advertising measures).^37^ ‐ Firm implementation in the training of practitioners.^37^ ‐ Training.^38^, ^39^ ‐ Tools for collecting and documenting (social) anamnesis.^48^ ‐ Patients questionnaires as tools for patients preferences.^48^
*'Knowledge (including knowledge of condition/scientific rationale)' construct* ‐ Content of CPGs was not part of education.^37^ ‐ Deficient physician training.^35^ ‐ Not enough knowledge considering the usage of biologicals.^52^ ‐ Do not know the CPG(s).^40^, ^46^, ^50^, ^53^, ^54^	*'Knowledge (including knowledge of condition/scientific rationale)' construct* ‐ Physician education.^35^ ‐ Workshops/conferences for practitioners about the content of CPGs.^37^
Skills	
*'Skills development' construct* ‐ No CPG‐training.^37^, ^50^ ‐ Lack of experiences.^54^ ‐ Limited experience (with treatment).^52^, ^56^ ‐ Lack of skills.^54^ ‐ Not familiar with the use of CPGs.^37^ ‐ The number of patients treated with moderate to severe psoriasis.^52^	*'Skills development' construct* ‐ Provision of instruction how to implement a CPG into practice.^54^ ‐ A manual that transfer the international CPGs in everyday practice.^38^, ^39^
*'Practice' construct* ‐ Inexperience (prescribing sacubitril/valsartan combination or ranolazine).^36^ ‐ Not enough experience (with treatment).^52^ ‐ Years of professional experience.^52^, ^53^ ‐ They learned digitally guided biopsy in their training when there was no sonographically guided method.^55^ ‐ Not proficient in digitally guided technology.^55^ ‐ (necessary pretesting) Is too complex.^52^	*'Practice' construct* ‐ Frequency of contact to patients of interest.^40^
	*'Competence' construct* ‐ Doctors general organisational skills.^54^
Social and personal role and identity	
*'Group/social identity/leadership' construct* ‐ Patient‐physician relationship.^35^ ‐ Communicative barriers.^48^, ^50^	*'Group/social identity/leadership' construct* ‐ CPGs that enhance the practitioner's credibility towards patients.^54^ ‐ Patients‐communication.^50^ ‐ Maintain regular physician‐patient‐contacts.^50^
*'Professional identity/boundaries/role' construct* ‐ Perceived constraints of therapeutic freedom due to CPGs.^32^, ^37^ ‐ Motivation (CPG was seen as criticism of the PCPs' work).^54^ ‐ Working years spent in a dermatological hospital, which count as a deduction in clinical education or experience.^52^ ‐ Continuation of the medication initiated in the hospital (reasoning that doctors in the hospital are the absolute experts).^36^	*'Professional identity/boundaries/role' construct* ‐ Leaving adequate space to draw their own conclusions.^51^ ‐ Profession.^40^
Beliefs about capabilities	
*'Self‐confidence' construct* *‐* Deficient therapeutic self‐confidence.^35^	*'Self‐confidence' construct* ‐ Increased self‐confidence of the doctor.^54^
*'Perceived competence/perceived behavioural control' construct* ‐ A conventional systemic/photo/topic therapy is sufficient in most patients.^52^	
Optimism	
*Not applicable*	*Not applicable*
Beliefs about consequences	
*'Outcome expectancies' construct* ‐ CPGs are not helpful to improve patient care.^33^ ‐ The lack of efficiency.^52^ ‐ Did not see any necessity for statins in patients after MI with normal lipid values.^36^ ‐ A conventional systemic, photo or topic therapy is sufficient in most patients.^52^ ‐ Lack of outcome expectancy.^40^ ‐ CPGs contradict my own clinical expertise.^33^ ‐ CPGs are hindrances to my clinical decision‐making.^33^ *‐* Lack of evidence.^52^ ‐ Personal experience.^38^, ^39^, ^42^, ^43^, ^55^ ‐ Missing possibility of control in case of side effects and necessary dose adjustment.^34^ ‐ Mental disorders cannot be treated according to the same pattern.^37^	*'Outcome expectancies' construct* *‐* Treatment success.^54^
*'Beliefs' construct* ‐ Physician disapproval of CPGs.^35^ ‐ Disagreement with recommendations (whole or partially).^37^, ^53^ ‐ Unrealistic.^53^ ‐ Not useful at all.^46^ ‐ I have doubts regarding the safety of biologicals.^52^ ‐ Perceived evidence‐base of the CPG.^54^ ‐ Patients would be confused by detailed education.^55^ ‐ Duration of the treatment.^56^ ‐ The expectation of a negative test result.^49^ ‐ The PD‐L1 status was not decisive for the therapy.^49^ ‐ The risk/benefit profile is not adequate.^52^ ‐ Tumour sample was not suitable for PD‐L1 testing.^49^	*'Beliefs' construct* ‐ The personal conviction about the benefit of the guideline.^47^ ‐ The CPG simplifies the practice.^54^
Reinforcement	
*'Consequents' construct* ‐ For syst. corticosteroids: rebound phenomenon after discontinuation of syst. Corticosteroids.^56^ ‐ Nonbinding nature of CPG adherence.^37^	*'Consequents' construct* ‐ Creation of commitment (e.g. evaluation by superior authority).^37^ ‐ Acknowledged the CPG being a good starting point for their self‐study.^51^
*'Incentives/reinforcements/punishments' construct* ‐ Low reimbursement (dermatology).^52^ ‐ Unfavourable cost‐effort profile.^56^ ‐ Economic disadvantages if the recommendation is implemented.^34^	*'Incentives/reinforcements/punishments' construct* ‐ Reduced budget when not following CPG recommendations.^37^ ‐ CPGs that are financially supported.^54^ ‐ Offered protection in case of legal responsibility.^54^
*'Contingencies' construct* ‐ Concern about possible recourses.^34^, ^52^	
Intentions	
*'Stages of change models' construct* ‐ General rejection of guidelines.^37^ ‐ General reluctance to work according to protocols.^51^ ‐ Lack of motivation.^40^	
Goals	
*'Goals priorities' construct* ‐ Competing priorities with other diseases being perceived as more relevant.^54^ ‐ A need for other, more important drugs which cannot be given with recommended medication.^36^	*'Goals priorities' construct* ‐ Patients questionnaires as tools for patients preferences.^48^
Memory, attention and decision processes	
*'Decision making' construct* ‐ Missing evidence for priorisation.^48^	*'Decision making' construct* ‐ Patients involvement in decision‐making.^36^ ‐ Priorisations of medications.^48^ ‐ Tools to prioritize of diseases (prognostically).^48^ ‐ Tools for considering common comorbidities.^48^ ‐ Good to weigh the wishes of the patients.^51^
*'Memory' construct* ‐ One own experiences (own CPGs in mind).^50^	
Environmental context and resources	
*'Organisational culture/climate (micro‐, meso‐, macrosystem)' construct* ‐ Organisational barriers.^45^ ‐ Using CPGs is not supported at my workplace.^33^ ‐ Support of the practice team.^54^ ‐ Employer rejects CPG recommendations.^37^ ‐ Fragmentation of supply system (e.g. lack of cooperation with other professions).^37^, ^51^ ‐ Deficient cross‐sectional coordination.^35^ ‐ Cost coverage by health insurance.^56^ ‐ Health care system deficiencies.^35^ ‐ Lack of regional availability of interventions recommended (e.g. echocardiology in rural areas).^37^, ^50^ ‐ No pharmacy for continuous care.^38^, ^39^ ‐ No PE availability on weekends.^38^, ^39^ ‐ Missing health care structures.^38^, ^39^ ‐ Physical environment.^54^ ‐ Non‐alignment of CPGs and reimbursement.^35^ ‐ Ecconomic reasons.^42^, ^43^ ‐ Economic disadvantages if the recommendation is implemented.^34^ ‐ The costs of topical treatment for patients.^56^ ‐ Impact on budget (due to prescription).^56^ ‐ Inability to prescribe over the counter medicaments.^56^ ‐ Insufficient opportunities for patient education.^56^	*'Organisational culture/climate (micro‐, meso‐, macrosystem)' construct* ‐ Communication with outpatient physicians.^42^, ^43^ ‐ Involvement of other professions.^51^ ‐ Cross‐provider collaboration.^35^ ‐ Networking between multi‐professional teams (e.g. concerning additional interventions like occupational therapy).^37^ ‐ Accompanying remote support in diagnosis for difficult cases.^54^ ‐ Early interdisciplinary, e.g. cardiology, psychotherapy.^50^ ‐ Tertiary care centres.^54^ ‐ Adequate staff numbers.^54^ ‐ Certification of the centres.^38^, ^39^ ‐ Alignment of CPG and reimbursement.^35^ ‐ Consideration from CPG, which medications are available or reimbursable in Germany.^42^, ^43^
*'(human) Resources/material resources' construct* ‐ The availability of end‐effector or exoskeleton‐based devices and treadmills.^51^ ‐ Limited access to CPG.^37^ ‐ No access to CPG with high evidence.^50^ ‐ Lack of financial resources.^37^ ‐ A need for financial compensation.^51^ ‐ Costs of therapy.^45^, ^52^, ^56^ ‐ Cost considerations.^49^ ‐ Missing clinical information at the time of therapy decision.^35^	*'(human) Resources/material resources' construct* ‐ Electronic therapy decision support.^35^ ‐ Real‐time deviation tracking.^35^ ‐ Appointment reminder system.^35^ ‐ An interactive software.^38^, ^39^ ‐ Notifications to practitioners when publishing a new CPG.^37^ ‐ The journal where the CPG is published.^42^, ^43^ ‐ Electronic availability of knowledge resources.^54^ ‐ Patient ID or other info‐ materials.^50^ ‐ Implementing procedures into patient‐management softwares.^50^ ‐ Financial support/concepts for CPG development and implementation (e.g. model projects).^37^, ^54^
*'Environmental stressors' construct* ‐ High workload^2^, ^12^, ^50^ ‐ Lack of time.^32^, ^37^, ^42^, ^43^, ^46^, ^50^, ^51^, ^52^, ^53^, ^55^, ^56^ ‐ No time to read CPGs.^33^ ‐ Duration for counselling.^56^ ‐ The duration until test result.^21^ ‐ Dissatisfaction with time‐money‐relation.^50^ ‐ Too many CPGs.^53^ ‐ Organisational aspects.^32^ ‐ Different statements from different CPGs of different medical societies for the same topic.^32^	
*'Salient events/critical incidents' construct* ‐ Local (antibiotic) resistance.^42^, ^43^ ‐ Information by the media beforehand.^55^	*'Salient events/critical incidents' construct* ‐ Information by the media beforehand.^55^ ‐ Inclusion of family members.^2^
	*'Person‐environment interaction' construct* ‐ Public relations.^50^ ‐ More visibly presented to the public.^42^, ^43^
Social influences	
*'Social support' construct* ‐ Exchange with colleagues.^54^ ‐ Colleagues do not support this treatment.^52^	*'Social support' construct* ‐ Social facilitators.^54^ ‐ Communication with outpatient physicians.^42^, ^43^
*'Power/intergroup conflict' construct* ‐ Influences by the interests of pharmaceutical sales representatives (misinformation of the physician).^50^	
	*'Social norms' construct* ‐ Normative influences.^47^
Emotions	
*'Fear' constructs* ‐ Fear to try something new.^37^ ‐ Unconfident to comply according to CPGs.^52^	
Behavioral regulations	
*'Action planning' construct* ‐ Clinicians are following internal protocols.^44^	*'Action planning' construct* ‐ Consequent, well‐structured, clear guidance/instruction of the patient.^50^ ‐ Simple explanation of disease and treatment with a repetition of the relevant parameters (towards patient).^50^
*'Breaking habit' construct* ‐ Problems changing routines.^51^ ‐ Idleness (inertia).^50^	*'Breaking habit' construct* ‐ Doctors self‐discipline.^54^
Guideline characteristics	
*'Transfer'* ‐ Lack of general practical relevance.^37^, ^45^, ^46^ ‐ Lack of relevance for my professional practice.^37^ ‐ Poorly adapted to the everyday reality of the primary care setting.^51^, ^54^ ‐ Too theoretical.^44^ ‐ Not practicable.^50^ ‐ Inappropriate form of publication.^37^	*'Transfer'* ‐ Adjustment to the reality/circumstances of primary care.^54^ ‐ Considering which medications are available/reimbursable in Germany.^42^, ^43^ ‐ Consideration of local resistance statistics.^42^, ^43^ ‐ Consideration of specific patient subgroups.^54^ ‐ Integrating the 'Multimorbidity' in subject‐specific guidelines.^48^ ‐ Greater involvement in the development of guidelines (e.g. practitioners, from setting with lower resources, patients).^37^, ^54^ ‐ Tools for involving the practice team in the case management process.^54^ ‐ Presentation of care pathways.^56^ ‐ Feasibility/implementability.^56^
*'Format'* ‐ Too much effort (too long versions).^32^, ^37^, ^46^, ^54^ ‐ Organisation of the CPG.^42^, ^43^ ‐ No user‐friendly format.^32^ ‐ CPGs are frequently unclear.^54^ ‐ CPGs are confusing.^53^ ‐ CPGs are too complex.^44^	*'Format'* ‐ Tools for interactive electronic CPGs.^48^ ‐ Wording/easier language.^37^, ^56^ ‐ Shorter Versions (e.g. practical aids, checklists, 1‐page summaries).^37^, ^42^, ^43^, ^54^ ‐ Diagnostic/therapeutic algorithms.^54^ ‐ Layout/structure.^51^, ^56^ ‐ Layout/presentation.^54^, ^56^ ‐ Implementation materials e.g. slides.^56^ ‐ Type of CPGs.^38^, ^39^ ‐ Chapters in easy language.^42^, ^43^
*'Conflict of interest/transparency'* ‐ Editorial independence from other specialities.^54^ ‐ Editorial independence from pharmaceutical industry.^54^ ‐ No transparency of CPGs development.^42^, ^43^	*'Conflict of interest/transparency'* *‐* Competent, independent updates.^50^
*'Evidence base/up‐to‐dateness'* ‐ Inconsistent recommendations.^37^ ‐ Not robust scientific evidence.^44^ ‐ Different statements from different CPGs from different medical societies for the same topic.^32^ ‐ I think parts of the CPG are incorrect.^51^	*'Evidence base/up‐to‐dateness'* ‐ Scope of background text.^56^ ‐ Clear presentation of evidence.^56^ ‐ CPGs Revision.^35^
*'Missing information'* ‐ No recommendations for complicating factors (e.g. side effects, comorbidities, polypharmacy).^36^ ‐ Consideration of specific patient sub‐groups (older patients).^54^ ‐ Insufficient info in patient education.^56^ ‐ Insufficient info on emollients.^56^ ‐ Can't find in the needed information.^32^	*'Missing information'* ‐ CPGs for high‐prevalence diseases.^54^ ‐ Presentation and commentary of the elaborated cases.^48^ ‐ Patient information.^42^, ^43^, ^54^ ‐ Self‐help materials in CPGs.^54^

Abbreviations: CPG, clinical practice guidelines; TDF, Theoretical Domains Framework.

The subsequent sections elaborate on all identified barriers and facilitators, categorized into the 14 TDF domains. These domains are presented in descending order based on the quantity of identified barriers and facilitators, from the one with the highest to the one with the lowest number. At the end, the additional domain *guideline characteristics* is described.

#### 3.3.1 Environmental context and resources

The identified barriers in this domain are related to the following theoretical constructs: ‘organizational culture and climate,’ ‘material and human resources,’ ‘environmental stressors,’ ‘salient and critical events,’ and ‘person ‐ environment interaction.’

Barriers associated with the ‘organizational culture and climate’ construct included issues of cooperation and coordination among professionals, healthcare deficiencies, and economic disadvantages. For instance, challenges such as the non‐cooperation of colleagues, doctors, and managers in utilizing CPGs (also referred to as the fragmentation of the supply system) were highlighted.^37^, ^51^ Organizational issues^45^ in workplace,^33^ in practice teams^54^ and cross‐sectional coordination^35^ were also identified. Barriers in this context encompassed health care system deficiencies,^35^ nonavailability of health care structures overall (e.g. pharmacy for continuous care) or at different time points (e.g. on weekends)^38^, ^39^; lack of regional availability of interventions recommended in CPGs,^37^, ^50^ and issues related to reimbursement from health insurances^35^, ^56^ The study by Traidl et al. provides a detailed description of the economic disadvantages of adherence to CPGs,^56^ including treatment costs borne by patient when adhering CPGs, impact on doctors' budgets and restrictions on prescribing over‐the‐counter medicines. Identified facilitators included communication with colleagues or other professions,^35^, ^37^, ^42^, ^43^, ^50^, ^51^ remote support for difficult cases,^54^ staff numbers,^54^ certification of the centers,^38^, ^39^, ^54^ alignment of the reimbursement system and CPGs, and consideration of medications that are available or reimbursable in Germany.^42^, ^43^

Factors categorized under the ‘material and human resources’ construct included the availability of diagnostic devices,^51^ limited access to CPGs,^37^ insufficient financial resources and compensation,^37^, ^51^ and cost considerations.^45^, ^49^, ^52^, ^56^ Facilitators addressed the electronic therapy support,^35^, ^50^, ^54^ real‐time tracking,^35^ interactive software,^38^, ^39^ notifications when CPGs are published,^37^ patient management information,^50^ and financial support concepts.^37^, ^54^

The ‘environmental stressors’ construct included high workload,^35^, ^50^ lack of time,^32^, ^33^, ^37^, ^42^, ^43^, ^46^, ^50^, ^51^, ^52^, ^53^, ^55^, ^56^ organizational aspects,^49^ dissatisfaction with the time‐money relation,^50^ and confusing statements in different CPGs.^32^ No facilitators were identified here.

In the ‘salient and critical events’ construct, treatment's resistance (e.g. local antibiotic resistance)^42^, ^43^ was considered a barrier. Facilitating factors included the inclusion of family members^50^ and making CPGs visible to the public.^42^, ^43^ Pre‐information of patients by media^55^ was considered both a facilitator and a barrier to CPG adherence.

#### 3.3.2 Beliefs about consequences

Influencing factors from the theoretical constructs of ‘outcome expectancies’ and ‘beliefs’ were categorized here. Lack of efficiency of CPG according to doctors, lack of evidence, and personal experiences were seen as hindrances to doctors clinical decision‐making.^33^, ^36^, ^52^ For instance, physical therapists (PTs) reported that CPGs did not help improve patient care,^33^ and general practitioners (GPs) stated that fewer tests than suggested by CPGs are sufficient for most patients.^36^ Facilitators encompassed instances of treatment success when following CPGs.^54^

Practitioners reported varying beliefs about working with CPGs. In some studies, CPGs were perceived as unrealistic, some physicians disagreed fully or partially with the CPGs.^35^, ^37^, ^52^, ^53^, ^54^ Many others expressed personal conviction and beliefs that CPGs simplify their practice.^47^, ^54^

#### 3.3.3 Knowledge

In the ‘task environment’ construct, identified barriers included clinicians' or other colleagues' lack of knowledge regarding the availability of CPGs,^33^, ^42^, ^43^ and the availability of CPGs.^50^ Facilitators included sources such as journals where CPGs can be found^42^, ^43^ and increased knowledge from the doctors.^54^

Barriers related to the ‘procedural knowledge’ construct included complexity, required knowledge, unawareness of certain CPGs, and difficulty in keeping track of all recommendations.^36^, ^51^ For example, PTs were unaware of the link between the CPGs and the phases of neuro‐rehabilitation.^51^ Facilitators included advertising measures to increase awareness about the advantages of CPGs,^37^ firm implementation,^37^ trainings,^38^, ^39^ tools to support anamnesis,^48^ and tools for considering patient preferences.^48^

Collected barriers regarding the ‘scientific rationale’ construct primarily included insufficient training of professionals for CPGs and their content.^35^, ^37^, ^52^ Facilitators encompassed education and/or workshops for practitioners about content of CPGs.^35^, ^37^

#### 3.3.4 Skills

Most barriers in this domain pertain to the ‘practice’ construct. Some dermatologists reported that a lack of experience with treatments (specifically biologicals) influenced adherence to CPGs.^52^ The working years impacted CPG adherence in dermatology and palliative care (longer working years was associated with higher competence to follow CPGs recommendations).^40^, ^52^ Additional barriers included alternative learning methods trained during education, a lack of proficiency in digitally guided technology, and inexperience with treatments.^36^, ^52^, ^55^ Frequency of contact with patients was considered as a facilitator.^40^

Barriers that corresponded to the ‘skills development’ construct included the non‐familiarity with the use of CPGs^37^ and little experience with patients in that condition/diagnosis.^37^, ^50^, ^52^, ^54^, ^56^ The provision of instructions, such as manuals that transfer CPGs to everyday practice^38^, ^39^, ^54^ was considered a facilitator.

#### 3.3.5 Reinforcement

The nonbinding nature of CPGs was reported to influence adherence,^37^, ^56^ while the creation of commitment (e.g. evaluation from superior authorities)^37^, ^56^ and acknowledgement that CPGs are a starting point for self‐study^51^ were seen as facilitators. Current reinforcement strategies were also seen as barriers. For example, low reimbursement,^52^ an unfavorable cost‐effort profile,^56^ and economic disadvantages when implementing CPGs.^34^ Facilitators included reduced budget when not following CPGs,^37^ financially supported CPGs, and protection in case of legal responsibility.^54^

#### 3.3.6 Social and personal role and identity

In the ‘group, social identity or leadership’ construct, communication (e.g. with patients) was perceived as both a barrier and a facilitator. Facilitators included maintenance of regular physician‐patient contacts^50^ and CPGs that enhance practitioners' credibility towards patients.^54^

Additional barriers in this domain included perceived constraints of therapeutic freedom from CPGs,^32^, ^37^ motivation,^54^ working years in the hospital,^52^ and continuation of medications initiated in the hospital.^36^ Facilitators included profession^40^ and CPGs that leave space for drawing own conclusions.^51^

#### 3.3.7 Memory, attention and decision processes

The identified barriers in this domain were the missing prioritization (based on which one could ultimately weigh the importance of individual diseases or medications),^48^ and ‘own guidelines’ in mind.^50^ Facilitators were tools or CPGs that prioritize medications, diseases, or comorbidities^48^ and patients' involvement in decision‐making.^36^, ^51^

#### 3.3.8 Social influences

Communication with colleagues and/or other professionals was considered both a barrier and a facilitator.^42^, ^43^, ^52^, ^54^ Influences of the industry (such as pharmaceutical industries) was also mentioned by Peter‐Klimm et al. (focus in general practitioners in CPGs for heart failure).^50^ In this regard, Lohmann et al. reported that normative influences were seen as facilitators in CPG adherence (topic: dementia).^47^

#### 3.3.9 Behavioral regulations

Barriers included adherence to internal protocols rather than CPGs,^44^ inertia^51^ and routine.^50^ Facilitators included understandable instruction of the patients'^50^ and doctors' self‐discipline.^54^

#### 3.3.10 Intentions

General rejection or reluctance to work according to protocols was identified as a barrier to CPG adherence.^37^, ^51^ No facilitators were categorized in this domain.

#### 3.3.11 Beliefs about capabilities

The therapeutic self‐confidence of physicians was considered both a barrier^35^ and a facilitator.^54^ The belief that certain therapies are sufficient for treating most patients was reported to hinder the CPG adherence.^52^

#### 3.3.12 Goals

Scheffler et al. reported that the prioritization of other diseases might be a barrier to following the rehabilitation CPG after stroke.^51^ In the study by Freier et al., GPs expressed the need to prescribe what they considered to be ‘more important medications’ than those listed in the CPGs for the treatment of myocardial infarction.^36^ The implementation of questionnaires as tools for measuring patients' preferences was considered a facilitator.^48^

#### 3.3.13 Emotions

Barriers related to the ‘fear’ construct were identified in this domain (namely, the fear of professionals to try something new^37^ or feeling unconfident to comply according to CPGs^52^). No identified facilitators were categorized in this domain.

### 3.4 Guideline characteristics in addition to the TDF domains

The barriers and facilitators in this domain pertain to various aspects, including the format of the CPGs, transfer, transparency/conflict of interest, evidence‐based, and missing information.

Critiques concerning the format of CPGs encompassed issues such as excessively lengthy versions,^32^, ^37^, ^46^, ^54^ organization,^42^, ^43^ lack of user‐friendly formats,^32^ and unclear/confusing or too complex CPGs.^44^, ^53^, ^54^ Identified facilitators included the use of easier language (wording), easy layout/structure, presentation of CPGs, implementation materials (e.g. slides),^56^ tools for interactive electronic CPGs,^48^ shorter versions^37^, ^42^, ^43^, ^54^ and algorithms.^54^

Barriers associated with knowledge transfer included an inappropriate form of publication,^37^ a lack of practical relevance in general^37^, ^45^, ^46^ or for specific professions,^37^ poor adaptation to everyday reality (in primary care setting),^51^, ^54^ and too theoretical/impracticable CPGs.^44^, ^50^ Facilitative strategies involved adjusting to the clinical reality, considering patient subgroups, involving practice teams in the case management process, greater clinician involvement in the CPGs development,^37^, ^54^ consideration of available/reimbursable medications in Germany,^42^, ^43^ consideration of local resistance statistics,^42^, ^43^ integration of “multimorbidity” in CPGs,^48^ and presentation of care pathways.^56^

Conflicts of interest, particularly editorial dependence of other specialties and pharmaceutical industries, and a lack of transparency in the CPGs development process, were considered as barriers.^42^, ^43^, ^54^ Facilitators included competent and independent updates of CPGs.^37^

The evidence‐base of CPGs was questioned in different studies due to inconsistent recommendations between CPGs,^32^, ^37^ no robust scientific evidence,^44^ incorrect parts in CPGs,^51^ and the scope of background text and presentation of evidence.^56^ Revisions were reported to facilitate adherence to CPGs.^35^

Lastly, studies reported the issue of missing information in some CPGs, for example insufficient information in patient education and emollients in the CPG for atopic dermatitis,^56^ difficulties in finding needed information in CPGs in general,^32^ concerning complicating factors,^36^ or the absence of consideration for patient subgroups.^54^ Facilitators included the availability of CPGs for high‐prevalence diseases, self‐help materials, and patient information.^42^, ^43^, ^54^ Additionally, doctors expressed a preference for presentations and commentaries on elaborated cases.^48^

### 3.5 Stakeholder involvement

The stakeholder meeting took place online in May 2023. Following the presentation of the results, stakeholders expressed interest in an international comparison of the findings. The discussion also covered topics such as prioritizing barriers and facilitators, and quantifying results. The discussion and comments provided during this meeting contributed to a critical reflection on the results.

## 4 DISCUSSION

This review provides information on barriers and facilitators to CPG adherence in Germany. Germany operates a complex and decentralized healthcare system, with governance shared between federal and state authorities, alongside corporatist entities for self‐governance.^58^ According to European Observatory on Health Systems and Policies, it classifies as social health insurance model, featuring private service providers and payers (insurance companies) and the Ministry of Health and its affiliated agencies as regulators.^59^ Collaboratively, AWMF and medical societies develop and disseminate the CPGs. Service providers themselves are responsible for providing the possibilities and resources needed for the implementation and adherence to these CPGs.

To our best knowledge, this is the first review that focuses on influencing factors to CPG adherence in Germany. At the international level, there are two reviews similar to ours. The umbrella review by Zhou et al. explores the barriers and facilitators to CPG implementation at the international level using the TDF and Behaviour change wheel.^60^ In their scoping review, Stewart et al. focus on exploring the theories that are used to investigate CPG adherence. Although not its primary aim, this scoping review provides information on the most commonly reported influencing factors from the studies identified.^61^ In Germany, we identified a recently published protocol of a scoping review that focuses on influencing factors to adherence to CPGs in physiotherapy.^62^

The results of our systematic review show that *environmental factors and resources*, *guideline characteristics*, *beliefs about consequences*, and *knowledge* were the most frequently reported factors that hinder practitioners to adhere to CPGs. The most reported facilitators were *environmental factors and resources*, *guideline characteristics*, and *reinforcement*.

Our results align with those of two comparable reviews that investigated strategies to enhance CPGs adherence. The scoping review by Stewart et al. identified *environmental context and resources* (e.g., outdated, burdensome CPGs, lack of resources), *beliefs about consequences* (e.g., scepticism about clinical gains from CPGs), and *knowledge* (e.g., the existence of CPGs) as key barriers to CPGs adherence.^61^ Similarly, the umbrella review by Zhou et al. highlighted *environmental context and resources* and *knowledge* as the most frequently reported barriers.^60^

While our's and Zhou et al.'s review share the abovementioned similarities, there are differences in the reported barriers. Zhou et al. listed *social influences* and *skills* among the most reported barriers, whereas in our review, *beliefs about consequences* emerged as the second most frequently reported barrier. Regarding facilitators, both our review and Zhou et al.'s identified the *environmental context and resources*, and *reinforcement* as the most frequently reported facilitators within TDF.

Given the substantial number of barriers and facilitators identified concerning the development and dissemination of CPGs, an additional domain, *guideline characteristics*, was incorporated. The barriers and facilitators in this domain closely paralleled the findings of the review by Bierbaum et al. (focus on cancer).^63^ Corresponding to our results, concerns regarding evidence (doubts on scientific evidence), CPG content (inconsistent recommendations and missing information), and clinician uncertainty (lack of clinical‐relevance, conflicts of interests/transparency), were reported in both studies. Bierbaum et al. also identified facilitators to CPG adherence, such as accessibility and ease of use, endorsement, dissemination; awareness, and belief in the relevance of CPGs. Similar facilitators emerged in our review, specifically, strategies to enhance CPGs use (format, layout, language, patient materials etc), transfer strategies (e.g. adjustments to clinical reality, reimbursement system etc), and efforts to raise awareness about the evidence base of CPGs, and minimizing conflicts of interests. These overlapping facilitators highlight the significance of key elements in fostering successful adherence to CPGs.

Research conducted in different countries indicates that different health professionals might encounter different barriers and facilitators for CPG adherence. For instance, a study from Denmark relieved that personal identity of professionals' influence CPG adherence.^64^ Similarly, an Australian study identified differences among individual health profession groups.^65^ We were unable to explore this important question due to limited information at this stage. Only two of the included studies in this review described changes in barriers and facilitators of CPGs adherence among different professions,^37^, ^42^, ^43^ thus preventing a comprehensive analysis.

### 4.1 Strengths and limitations

We actively engaged stakeholders throughout most of the phases of research. According to Keown et al, stakeholder involvement in systematic reviews entails potential benefits in terms of relevance and clarity of findings and improvement in the dissemination process.^66^ Additionally, we conducted a thorough literature search aimed at identifying all relevant studies.

However, some limitations need to be considered. First, our review concentrated on CPG adherence rather than just CPG implementation. We collaboratively defined ‘guideline adherence’ with involved stakeholders as *“the consistency between clinicians' actions and recommendation of the CPGs for cases where deviations from CPG recommendations are not justified”*. This definition is similar to the one provided by Gardner.^67^ It is crucial to note, however, that the included studies in our review did not consistently provide definitions for terms they used (adherence, implementation, uptake, etc.). As a result, we included primary studies that used alternative terms (e.g. compliance, implementation, uptake, execution, etc.) when they actually referred to adherence, as defined above.

Our results may be constrained by the data collection in the included articles. Among the 24 studies in our review, only eight collected data on barriers and/or facilitators though open‐ended questions. Others utilized closed‐ended questions (i.e. multiple‐choice statements), or analyzed participants' sociodemographic data alongside their CPGs adherence level. Therefore, there is a risk of detection bias for the included studies, which might also impact the number and categories of identified barriers and facilitators in our review.

Lastly, there is a need for further exploration of the barriers and facilitators specific to CPGs in Germany in specific health condition, professions, and settings. We strongly advocate for future studies in this field to focus on the regional long‐term compliance/adherence of existing CPG.

### 4.2 Implications of our findings

Different clinical settings across various regions in Germany exhibit variations in the availability of resources, diagnostic and treatment tools, and collaboration among professionals.^37^, ^50^ Therefore, we suggest the inclusion of different perspectives from various settings and regions within Germany (e.g., through consultation methods) during the CPG development process to account for different settings.

Since *knowledge* was often identified as barrier, guideline development teams should focus on appropriate dissemination and implementation strategies addressing all relevant professions and their different practice preferences. Moreover, they need to seek further expertize with regard to dissemination strategies (preferred strategies were workshops, support team, education curricula, and software‐applications that notify the publication of new or updated CPGs). Additionally, patient versions for each CPG and other patient‐directed CPG‐based knowledge tools might facilitate the clinician‐patient collaboration.^68^

Outpatient practices, hospitals, university clinics, and clinical federations, such as the German Hospital Federation (DKG) should play an active role in transforming the culture and processes within their settings to promote greater utilization of CPGs. Emphasis should be placed on enhancing communication within teams, across teams, and ensuring convenient access to CPGs at the point of care delivery. CPGs should be accessible at the point of care, for example via practice or hospital information systems. Additionally, these entities need to provide the necessary infrastructure to support implementation strategies, including training and workshop for clinicians for CPG content. In addition, continued efforts, including certification of medical institutions and quality management systems, are essential to ensure their commitment to following CPGs by healthcare personnel.

Consequences of recommendations in CPGs for reimbursement should be discussed transparently. Preferred incentives that facilitate the use of CPG in clinical practice include financial benefits for clinicians when following CPGs. However, as intended deviations from CPG recommendations will always be allowed, financial incentives would also come along with drawbacks and might even worsen the quality of health care.^69^ Therefore, adherence to CPGs can only be one out of many quality indicators. This will have implications when designing pay‐for‐performance programs, for example. In addition, the availability of decision support systems (computerised or support by other peer‐professionals) to assist the use CPG should be considered for an increased adherence of CPGs. We strongly support the conversion of CPG recommendations into practical clinical pathways, particularly within Disease Management Programs (DMPs). This integration represents a powerful instrument for effectively implementing CPGs in clinical practice. Establishing DMPs based on high quality CPGs is an important example of how CPGs and provision of health care may be linked closely. DMPs can contribute to implementing CPG and subsequently to improving clinical outcomes.^70^

## 5 CONCLUSION

In this systematic review we investigated the barriers and facilitators of adherence to CPGs in Germany. The three most frequently reported barriers were associated with environmental factors and resources, beliefs about consequences, and knowledge. The most commonly reported facilitators encompassed environmental factors and resources, beliefs about consequences, and reinforcement. Notably, a high number of identified barriers and facilitators were related to CPGs themselves, including aspects such as format and development process.

The comprehensive identification of reported barriers and facilitators within each TDF domain, which itself derives from the behaviour change theories, holds the potential to assist researchers and clinicians in promoting and enhancing utilisation of CPGs in clinical practice. The insights gained from this systematic review serve as a foundation for the development of future strategies involving various actors in the CPG development or implementation process.

## CONFLICT OF INTEREST STATEMENT

The authors declare no conflict of interest.

## Supporting information

Supporting information.

## Data Availability

The data that support the findings of this study are available from the corresponding author upon reasonable request.
