# Supplementary material for: Barriers and facilitators of adherence to clinical practice guidelines in Germany—A systematic review
Source: J Eval Clin Pract. 2024 Oct 16;31(3):e14173. doi: 10.1111/jep.14173 (PMC12021332; doi:10.1111/jep.14173)
Supplement: Supplementary file 1 — Supporting information. [file JEP-31-0-s001.docx]

**Supplementary materials**

**Shehu et al.: Barriers and facilitators of adherence to clinical practice guidelines in Germany - a systematic review**

Table of content

**Supplementary material 1: Electronic search terms and syntax2**

Databases2

Medline via PubMed search strings2

Google scholar search strings2

**Supplementary material 2: Quality assessment of included studies via Mixed Methods Appraisal Tool3**

Table 1: Methodological quality criteria for qualitative studies3

Table 2: Methodological quality criteria for quantitative studies4

Table 3: Methodological quality criteria for mixed-methods studies5

## Supplementary material 1: Electronic seach terms and syntax

# Databases

### Medline via PubMed search strings.

#1 (german* [Title/Abstract] OR "deutsch*"[Title/Abstract] OR "deutsch*"[Transliterated Title] OR "german*"[Affiliation] OR "German"[Language])

#2 ("enabling"[Title/Abstract] OR "enable*"[Title/Abstract] OR "barrier*"[Title/Abstract] OR "obstacle*"[Title/Abstract] OR "facilitat*"[Title/Abstract] OR "hinder*"[Title/Abstract] OR "influenc*"[Title/Abstract])

#3 ("guidelines as topic"[MeSH Terms] OR "guideline*"[Title/Abstract])

#4 ("patient compliance"[MeSH Terms] OR "compliance"[MeSH Terms] OR "complian*"[Title/Abstract] OR "adheren*"[Title/Abstract] OR "implement*"[Title/Abstract] OR "guideline adherence"[MeSH Terms] OR "practicab*"[Title/Abstract])

#5 2011/01/01:3000/12/31[Date - Publication].

#1 AND #2 AND #3 AND #4 AND #5

### Google scholar search string:

Clinical guidelines AND Adherence AND Germany

klinische Leitlinien Adhärenz

## Supplementary material 2: Quality assessment of included studies via Mixed Methods Appraisal Tool

# Table 1: Methodological quality criteria for qualitative studies

| **Methodological quality criteria**  ***(qualitative studies)*** | | AZQ 2021^+^ | Freier 2020 | Mühlhäuser 2018 | Peters-Klimm 2012 | Stephan 2018 | Tiedje 2017 |
| --- | --- | --- | --- | --- | --- | --- | --- |
| *Screening questions (for all types)** | | | | | | | |
| S1 | Are there clear research questions? | Yes | Yes | No | Yes | Yes | Yes |
| S2 | Do the collected data allow to address the research questions? | Cnt | yes | No | Yes | Yes | Yes |
| *Questions for qualitative study designs* | | | | | | | |
| 1 | Is the qualitative approach appropriate to answer the research question? |  | Yes | Yes | Yes | Yes | Yes |
| 2 | Are the qualitative data collection methods adequate to address the research question? |  | Yes | Yes | Yes | Yes | Yes |
| 3 | Are the findings adequately derived from the data? |  | Yes | Yes | Yes | Yes | No |
| 4 | Is the interpretation of results sufficiently substantiated by data? |  | Yes | Cnt | Yes | Yes | No |
| 5 | Is there coherence between qualitative data sources, collection, analysis and interpretation? |  | Yes | Yes | Yes | Yes | No |

Cnt = Cannot tell

* *Further appraisal may not be feasible or appropriate when the answer is ‘No’ or ‘Can’t tell’ to one or both screening questions.*

*^+^An assessment of the qualitative data was not carried out, as the barriers and facilitators for the CPGs were only collected using quantitative methods.*

# Table 2: Methodological quality criteria for quantitative studies

| **Methodological quality criteria *(quantitative descriptive studies)*** | | AZQ 2021 | Bahns 2021 | Brenner 2021 | Gaigl 2021 | Hoffmann 2021 | Kalles 2018 | Karbach 2021 | Kranz 2019, 2021 | Lapillone 2013 | Ostermann 2022 | Schielein 2018 | Schmieder 2012 | Tradl 2021 | Bannow 2021 | Laux 2018 | Lech 2022 | Lohmann 2017 | Scheffler 2022 | Westhof 2013 |
| --- | --- | --- | --- | --- | --- | --- | --- | --- | --- | --- | --- | --- | --- | --- | --- | --- | --- | --- | --- | --- |
| *Screening questions (for all types)** | | | | | | | | | | | | | | | | | | | | |
| S1 | Are there clear research questions? | Yes | Yes | Yes | Yes | Yes | Yes | Yes | Yes | Yes | No | Yes | Yes | Yes | Yes | Yes | Yes | Yes | Yes | No |
| S2 | Do the collected data allow to address the research questions? | Cnt | Yes | Yes | Yes | Yes | Yes | Yes | Yes | Yes | Cnt | Yes | Yes | Yes | Yes | Yes | Yes | Yes | Yes | Cnt |
| *Questions for quantitative descriptive study designs* | | | | | | | | | | | | | | | | | | | | |
| 1 | Is the sampling strategy relevant to address the research question? | Yes | Yes | No | Yes | Yes | Yes | Yes | Yes | Yes | Cnt | Yes | Yes | No | Yes | Yes | Yes | Yes | Yes |  |
| 2 | Is the sample representative of the target population? | No | No | No | No | No | Yes | Yes | Yes | No | No | Yes | Yes | Yes | Yes | Cnt | No | No | No |  |
| 3 | Are the measurements appropriate? | Cnt | Yes | Yes | Yes | No | No | Yes | No | Cnt | No | Yes | Yes | No | Cnt | No | Cnt | Yes | Yes |  |
| 4 | Is the risk of nonresponse bias low? | No | No | No | No | No | No | No | No | No | No | No | No | No | No | No | No | No | No |  |
| 5 | Is the statistical analysis appropriate to answer the research question? | Yes | Yes | No | Yes | Yes | Yes | Yes | Yes | Yes | Yes | Yes | No | Yes | Yes | Yes | Yes | Yes | Yes |  |

Cnt = Cannot tell

* *Further appraisal may not be feasible or appropriate when the answer is ‘No’ or ‘Can’t tell’ to one or both screening questions.*

# Table 3: Methodological quality criteria for mixed-method studies

| **Methodological quality criteria**  ***(mixed-method studies)*** | | AZQ 2021 |
| --- | --- | --- |
| *Screening questions (for all types)** | | |
| S1 | Are there clear research questions? | Yes |
| S2 | Do the collected data allow to address the research questions? | Cnt |
| *Questions for mixed methods study designs* | | |
| 5.1 | Is there an adequate rationale for using a mixed methods design to address the research question? | Cnt |
| 5.2 | Are the different components of the study effectively integrated to answer the research question? | Yes |
| 5.3 | Are the outputs of the integration of qualitative and quantitative components adequately interpreted? | Yes |
| 5.4 | Are divergences and inconsistencies between quantitative and qualitative results adequately addressed? | No |
| 5.5 | Do the different components of the study adhere to the quality criteria of each tradition of the methods involved? | NA |

Cnt = Cannot tell

NA = Not applicable

* *Further appraisal may not be feasible or appropriate when the answer is ‘No’ or ‘Can’t tell’ to one or both screening questions.*
